# Age and Auditory Spatial Perception in Humans: Review of Behavioral Findings and Suggestions for Future Research

**DOI:** 10.3389/fpsyg.2022.831670

**Published:** 2022-02-16

**Authors:** Michael Keith Russell

**Affiliations:** Department of Psychology, Bellevue University, Bellevue, NE, United States

**Keywords:** age, spatial, methodology, ecology, perception

## Abstract

It has been well documented, and fairly well known, that concomitant with an increase in chronological age is a corresponding increase in sensory impairment. As most people realize, our hearing suffers as we get older; hence, the increased need for hearing aids. The first portion of the present paper is how the change in age apparently affects auditory judgments of sound source position. A summary of the literature evaluating the changes in the perception of sound source location and the perception of sound source motion as a function of chronological age is presented. The review is limited to empirical studies with behavioral findings involving humans. It is the view of the author that we have an immensely limited understanding of how chronological age affects perception of space when based on sound. In the latter part of the paper, discussion is given to how auditory spatial perception is traditionally conducted in the laboratory. Theoretically, beneficial reasons exist for conducting research in the manner it has been. Nonetheless, from an ecological perspective, the vast majority of previous research can be considered unnatural and greatly lacking in ecological validity. Suggestions for an alternative and more ecologically valid approach to the investigation of auditory spatial perception are proposed. It is believed an ecological approach to auditory spatial perception will enhance our understanding of the extent to which individuals perceive sound source location and how those perceptual judgments change with an increase in chronological age.

## Introduction

In the real world, events occur within our vicinity and empirical research suggests we are able to detect and correctly identify those events when relying on sound alone. Individuals are nearly perfect at identifying bouncing and breaking glass jars (Warren and Verbrugge, 1984). Individuals are also capable of using sound to determine the gender of the pedestrian (Li et al., 1991) and whether a pedestrian is approaching or withdrawing (e.g., Kozhevnikova and Zhukov, 1990). Using sound, individuals are highly capable at recognizing the filling of a vessel and whether a vessel was filled to the brim (Cabe and Pittenger, 2000). When relying solely on sound, individuals are capable of judging the size of unseen dropped rods (Carello et al., 1998) and wooden balls (Grassi, 2005; Grassi et al., 2013), the roughness of a surface (Lederman, 1979), the material composition of a plate (Giordano and McAdams, 2006), the hardness of a mallet (Freed, 1990), the elasticity of a bouncing ball (Warren et al., 1987), and the shape of a struck object (Kunkler-Peck and Turvey, 2000).

Aside from judging the properties of an object or actions that occurred nearby, individuals commonly determine the spatial position of unseen sound sources. When an unexpected sound occurs (e.g., a glass dropped into a hard surface), individuals naturally look in the direction of the objects that created the event. Furthermore, individuals often need to detect the location of an unseen sound-producing object in order to avoid injury and potentially death. Being able to merely detect the existence of a viciously barking dog, a chain saw, or a motor vehicle, for example, is insufficient. Individual also needs to be keenly aware of the position of the potentially damaging object as it relates to their position. It would also be advantageous for individuals to be able to determine if and how the spatial position between object and perceiver is changing. In order to avoid collision, a pedestrian must be able to accurately determine that an automobile is approaching and when it will arrive at the individual’s location, as has been examined in a number of studies (e.g., Yan et al., 2007; Pörschmann and Störig, 2009; Braly et al., 2021).

Using an egocentric frame of reference, the position of an object can be described in a variety of ways. An important task an observer could perform is lateralization. Here, an observer simply needs to determine whether a target is located to their right or left (relative to the midline of the observer’s body). A more precise method of evaluating the ability of individuals to locate an unseen sound-producing object is to ask them to report the object’s distance, azimuth, or elevation. Distance refers to the extent of space between observer and target. Azimuth refers to the left-right, lateral, or horizontal angle (measured in degrees) between a sound source and the median plane of the observer (i.e., the plane corresponding to the observer’s midline). A target at an azimuth of 0, 90, 180, and 270° represents a target located precisely ahead, to the right, behind, and to the left of the individual, respectively. Elevation (also referred to as altitude) refers to the up-down or vertical angle (also measured in degrees) between a sound source and the horizontal plane of the observer. A target at an elevation of 0, 90, 180, and 270° represents a target located precisely ahead, above, behind, and below the individual, respectively. A target located at the origin (0° azimuth and 0° elevation) is located at ear level and directly in front of the individual. It is also possible for researchers to measure spatial acuity by calculating the minimum audible angle, i.e., the smallest perceptually detectable difference in position of two sound sources. As can be expected, the greater the physical separation between two sound sources, the greater the ability to discriminate the sound sources (e.g., Hartmann and Rakerd, 1989a; Perrott and Saberi, 1990; Brimijoin and Akeroyd, 2014).

It is well known that the ability of individuals to localize an unseen sound source is dependent on the difference in sound reaching the two ears (i.e., interaural differences) and the shape of the pinna (e.g., Middlebrooks and Green, 1991; Carlile, 1996; Blauert, 1997). When a sound source is located either directly ahead (0° azimuth) or directly behind an individual (180° azimuth), the sound contacts both ears at the same time and with equal intensity (interaural differences are null). When a sound-producing object is off center, the sound contacts the closer ear sooner (interaural time difference) and with greater intensity (interaural level difference) in comparison with the more distant ear. Interaural time and level differences are known to increase with an increase in deviation from 0° and reach maximal levels when a sound source is located at 90° (directly to the right) or 270° (directly to the left). It is also well known that interaural differences are dependent on signal frequency. In short, interaural time differences are limited to low-frequency sounds (below 1,500 Hz) while interaural level differences are limited to high-frequency sounds (above 1,500 Hz). The pinna is also known to influence the ability of individuals to localize sound sources. Perceived sound position is a function of a sound’s spectral properties and those properties are influenced by the shape of the pinna and target azimuth and elevation. Not surprisingly, alterations of the pinna have been found to impact the ability of individuals to localize sounds in the horizontal (e.g., Musicant and Butler, 1984; Oldfield and Parker, 1984; Hofman et al., 1998) and vertical planes (e.g., Roffler and Butler, 1968; Oldfield and Parker, 1984; Hofman et al., 1998).

Possibly not surprising to the reader are the decrements in perceptual capabilities that are coincident with an increase in chronological age. With respect to vision, an increase in age is often accompanied by an increase in the hardening (presbyopia) and the opacity (cataracts) of the lens. Possibly less well known is the apparent negative impact of age on perceptual judgments involving other modalities. In brief, an increase in chronological age has been found to negatively impact olfactory perception (e.g., Doty et al., 2011; Zhang and Wang, 2017; Olofsson et al., 2021), haptic perception (e.g., Thompson et al., 1965; Kleinman and Brodzinsky, 1978; Norman et al., 2016), and gustatory perception (e.g., Kaneda et al., 2000; Murphy et al., 2002; Fukunaga et al., 2005).

A complex relationship exists between age and the perception of sound source location. As will become evident, an accurate understanding of the impact of chronological age on auditory spatial perception requires the consideration of numerous factors. One such factor is hearing loss. Hearing loss often coincides and becomes more severe with the advancement of age. For example, it is common for individuals to become increasingly less sensitive to high-frequency sounds with an increase in chronological age (e.g., Rodríguez Valiente et al., 2014), which are considered important for sound localization (e.g., Butler and Humanski, 1992; Best et al., 2005; Zonooz et al., 2019). However, it is not always the case that hearing loss occurs with an increase in age. A small number of studies have determined the correlation between age and hearing loss to be weak or insignificant (e.g., Abel and Hay, 1996; Neher et al., 2011; Buchholz and Best, 2020). In addition, health and environmental factors have the potential to impair an individual’s hearing (for a review see Jayakody et al., 2018). Thus, it is possible hearing impairment is a direct result of those factors and not age.

Two purposes exist with regard to the present paper. Initially, a summary of the research relating chronological age and auditory spatial perception will be provided. The literature reviewed has been limited to empirical studies with behavioral findings in humans.^1^ Discussion is further limited to empirical studies that treated age as an independent variable. Undoubtedly, hearing loss is common among older individuals. Nonetheless, it is not a certainty an individual’s hearing will deteriorate with age. Chronological age and hearing impairment are, in fact, discrete variables. The intent of the present paper was to examine the degree to which chronological age affects auditory spatial perception. To accomplish that task, it seemed prudent to consider age independently of any confounding variables. Thus, studies that treated age and hearing loss as a single variable were excluded. To forewarn the reader, each of the studies will be described in more detail than is typically presented in an empirical or review article. The inclusion of a greater than normal amount of information is necessary for it relates to the latter part of the paper. In the latter part of the paper, comparisons will be drawn between traditional laboratory investigations and real-world settings. Despite the advantages of conducting research in a particular manner (e.g., simple sounds, anechoic settings, and stationary observers), the possibility exists that the traditional approach to auditory spatial perception fails provide insight into how chronological age affects judgments of sound source location when they occur in natural settings. It is believed an ecologically based approach will yield findings that relate directly to how individuals of varying age perceive the spatial position of an unseen sound source under everyday circumstances. It is further believed that an ecologically based approach will provide information that enhances the scientific communities’ understanding of the abilities of aged individuals to locate sounds and that information can, in turn, be used to enhance the performance of aged individuals in real-world settings.

## Significant Effects of Age on Auditory Spatial Perception

Table 1 contains a brief summary of the characteristics of participants, design, and analysis(es) performed relative to each of the studies subsequently discussed.

**Table 1:** Summary of characteristics of participants, design, and analysis related to studies presented in the literature review.

References	Age groups (in years)	Sample size	Stimuli	Range of localization (in degrees)	Statistical measure (s)
Abel et al. (2000)	7 age groups: 10–19, 20–29, 30–39, 40–49, 50–59, 60–69, 70–81. Means and standard deviations were not provided.	16 per age group	Broadband noise. One-third-octave noise band centered on 0.5 kHz and 4 kHz. Duration = 300 ms	Horizontal plane: 15–165	ANOVA and Regression
Abel and Hay (1996)	3 age groups: Young-Normal (18–38), Old-Normal (41–58), Old-Hearing Impaired (42–73). Means and standard deviations were not provided.	*N* = 24 Young-Normal, *N* = 24 Old-Normal, *N* = 23 Old-Hearing Impaired	Broadband noise. One-third octave noise bands centered at 0.5 or 4 kHz. Duration = 300 ms.	Horizontal plane: 30 to 150	ANOVA
Addleman et al. (2019)	2 age groups: Younger (21–33), Older (58–78). Means and standard deviations were not provided.	*N* = 13 Younger, *N* = 12 Older	Pink noise bursts. Frequency range = 0.2–8 kHz. Duration = 200 ms.	Horizontal plane: 10 to 180	ANOVA
Briley and Summerfield (2014)	3 age groups: Young (*M* = 22.9, *SD* = 2.8), Younger-Old (*M* = 65.1, *SD* = 3.6), Older-Old (*M* = 76.8, *SD* = 2.5)	*N* = 6 Young, *N* = 6 Younger-Old, *N* = 5 Older-Old	Summed pure tone frequencies/pink noise. Frequency range = 0.1–5 kHz. Duration = 1,510 ms.	Horizontal plane: 0 to 75	Descriptive statistics
Brungart et al. (2017)	2 are groups: Normal Hearing (*M* = 31.9), Hearing Impaired (*M* = 54.7). Standard deviations were not provided.	*N* = 16 Normal Hearing, *N* = 20 Hearing Impaired	7 periodic chirp signals. Frequency range = 0.1–15 kHz. Stimulus duration either 250, 100, or 4,000 ms.	Horizontal plane = −150 to +150 Vertical plane = −28 to +28	Correlation and Regression
Dobreva et al. (2011)	3 age groups per experiment: Experiment 1: Young (19–41), Middle Age (45–66), Elderly (70–81). Experiment 2: Young (19–37), Middle Age (51–66), Elderly (71–81). Means and standard deviations were not provided.	Experiment 1: *N* = 19 Young, *N* = 11 Middle Age, *N* = 12 Elderly. Experiment 2: *N* = 8 Young, *N* = 7 Middle Age, *N* = 6 Elderly	Band-limited, flat spectrum, Gaussian noise bursts. Duration = 150 ms.	Horizontal plane = −60 to +60 Vertical plane = −25 to +25	ANOVA
Fink et al. (2005)	2 age groups: Younger (*M* = 25), Elderly (*M* = 61.7). Standard deviations were not provided.	*N* = 20 per group	2 sound signals: clicks or pure tones. Clicks were noise, rectangular pulses. Duration = 1 ms. Sinusoidal tones 0.8 and 1.2 kHz. Duration = 10 ms.	Not applicable. Temporal order (lateralization) task. Sounds presented *via* headphones.	ANOVA
Freigang et al. (2014)	2 age groups: Young (*M* = 24.1, *SD* = 2.3), Older (*M* = 68.1, *SD* = 5.5)	*N* = 22 Young, *N* = 53 Older	Narrowband noise centered at 0.5 (0.375–0.75 kHz) and 3.0 kHz (2.25–4.5 kHz). Duration = 500 ms.	Horizontal plane: −98 to +98	ANOVA, T-test, and Correlation
Kolodziejczyk and Szelag (2008)	3 age groups: Young (*M* = 22, *SD* = 1.1), Elderly (*M* = 66, *SD* = 0.7), Very Old (*M* = 101.1, *SD* = 0.11)	*N* = 17 Young, *N* = 18 Elderly, *N* = 11 Very Old	300 Hz square tones. Duration = 15 ms.	Not applicable. Temporal order (lateralization) task. Sounds presented *via* headphones.	ANOVA
Otte et al. (2013)	3 age groups: Children (*M* = 9.5, *SD* = 1.3), Young Adults (*M* = 24.9, *SD* = 4.9), Older Adults (*M* = 68.4, *SD* = 4.7)	*N* = 18 Children, *N* = 10 Young Adults, *N* = 14 Older Adults	Gaussian white noise high-pass filtered at 0.5 kHz or low-pass filtered at 5, 7, 11, or 20 kHz. Duration = 150 ms.	Horizontal plane = −75 to +75 Vertical plane = −55 to +55	T-test and Correlation
Rønne et al. (2016)	2 age groups: Normal Hearing (*M* = 39, *SD* = 11), Hearing Impaired (*M* = 64, *SD* = 15)	*N* = 13 Hearing Impaired, *N* = 11 Normal Hearing	White noiseband pass filtered to 0.4–16 kHz. Duration of stimulus not provided.	Horizontal plane: 0 to 120	ANOVA
Savel (2009)	Age treated as a continuous variable. 48 individuals between 18 and 48 (*M* = 31, *SD* = 10), 2 individuals 61 and 62 (*M* = 61.5, *SD* = 0.5)	*N* = 48 Young, *N* = 2 Older	Filitered noise 0.25–2 kHz. Duration = 50 ms.	Horizontal plane: −77 to +77	Correlation
Szymaszek et al. (2006)	2 age groups: Young (*M* = 24 yrs., 8 months), Elderly (*M* = 64 yrs., 6 mos.) Standard deviations were not provided.	*N* = 17 Young, *N* = 16 Elderly	“Clicks.” No further description provided. Duration = 1 ms.	Not applicable. Temporal order (lateralization) task. Sounds presented *via* headphones.	ANOVA and Newman–Keuls
Whitmer et al. (2014)	Age treated as a continuous variable. Median = 63. Means and standard deviations were not provided.	*N* = 35	0.1 kHz click train. Duration = 500 ms.	Horizontal plane: −30 to +30	T-test and Correlation
Whitmer et al. (2021)	Age treated as a continuous variable. No specific age information was provided. Based on Figure 1, it appears participants ranged in age from 20 to 70.	N = 28	Speech segments with either 5- or 10-kHz cutoff frequency. Duration >5,000 ms.	Horizontal plane: −90 to +90	ANOVA and Correlation

### Lateralization Perception

As mentioned previously, an important auditory location task an individual can perform is that of lateralization. Individuals need only determine whether a sound source is located to the right or left of the individual’s midline. In short, a decrease in the ability to lateralize sounds is associated with an increase in chronological age. Szymaszek et al. (2006) examined the ability of individuals to determine the order (left-right or right-left) of two sequentially presented clicks. The period of time between click presentations was systematically varied. Young (*M* = 24 yrs., 8 mos.) and elderly (*M* = 64 yrs., 6 mos.) participants with normal hearing were compared. Threshold for lateralization was defined as the minimal time period between stimulus presentations that permitted 75% correct order identification. The thresholds for elderly individuals were significantly greater than that for young individuals. The mean threshold for young individuals was 66 ms. For elderly individuals, it was 88 ms.

Fink et al. (2005) employed the same task and likewise compared young (*M* = 25 yrs.) and elderly (*M* = 61.7 yrs.) individuals. While an increase in age was again found to negatively affect lateralization perception, threshold differences between the two age groups were dependent on the stimulus (clicks or tones), the method used to calculate the threshold (staircase or maximum-likelihood performance), and test session (session 1, 2, and 3). On average, the thresholds of older individuals were significantly greater than those of younger individuals. For young participants, the threshold was approximately 50 ms for clicks and approximately 15 ms for tones. For older participants, the mean threshold was approximately 65 ms for both clicks and tones. The mean difference in thresholds between the two age groups was smaller for clicks than for tones: 14.9 and 48.45 ms, respectively. With regard to test session, when the stimulus was a click, older and younger participants differed only with regard to the third test session and only when the staircase method was used to calculate the threshold. When the stimulus was a tone, the threshold difference between the two age groups decreased notably with an increase in session. For tones, the threshold for older participants decreased with an increase in test session. Thresholds were approximately 80 ms for session 1, 60 ms for session 2, and 40 ms session 3. For young participants, threshold values were largely unaffected by test session and were less than 20 ms.

Kołodziejczyk and Szelag (2008) likewise presented participants with the task of determining the order of a pair of stimuli (square wave tones). In that study, the thresholds for three age groups were determined: young (*M* = 22 yrs.), elderly (*M* = 66 yrs.), and very old (*M* = 101 yrs., 1 mo.). Threshold differences between the three age groups were evident. The mean threshold for the young, elderly, and very old participants was 37, 60, and 191 ms, respectively. Significance was limited to the differences between the young and very old, and between the elderly and very old.

### Localization Perception

The ability to determine which side of the body a sound source resides can be considered a rudimentary perceptual skill. Individuals perceiving and acting in real-world settings need to accurately locate sound-producing objects. Thus, the accuracy with which individuals can determine the precise location of a sound source is more insightful. Generally speaking, the findings of numerous studies suggest sound localization ability decreases with an increase in chronological age. Abel et al. (2000) evaluated the ability of seven age groups (10–19, 20–29, 30–39, 40–49, 50–59, 60–69, and 70–81 yrs.) to identify which loudspeaker (out of 4 or 8) was the origin of a target stimulus. In every ANOVA conducted, age was a significant factor. As expected, localization performance decreased with an increase in age, particularly beyond the first three decades of life. Regression analyses revealed that age accounted for 12–26 percent of the variance in perceptual accuracy.

In a study by Dobreva et al. (2011), young (19–41 yrs.), middle aged (45–66 yrs.), and elderly (70–81 yrs. old) individuals were required to direct (*via* a joystick) a laser-LED beam at the perceived location of the sound stimulus. The ability of individuals to accurately perceive azimuth and elevation was determined. In terms of perceived azimuth, while the main effect of age was not significant, age was a significant variable when different sound stimuli were taken into consideration. The three age groups were highly similar when the sound was low-pass (0.1–1 kHz) or high-pass (3–20 kHz) noise. Differences between the age groups were striking when the sound was ultra-high-pass (10–20 kHz) noise. Young participants were significantly more accurate than both middle-aged and elderly participants. In terms of perceived elevation, the main effect of age was again found to be significant. An increase in age was associated with a decreased ability to judge sound source elevation. Generally speaking, the perceptual judgments of young individuals were the most accurate and the performance of middle-aged individuals tended to be more similar to the performance of elderly individuals than to young individuals.

Whitmer et al. (2014) positioned participants in the center of a cloth covering a 24-speaker array that ranged from −45 to +45°. From one of the speakers, a 100-Hz click train was presented. The task of the participant was to indicate on a monitor situated in front of them where they believe the sound originated. For each individual, localization precision (variability) and accuracy (absolute difference between mean perceived and actual location) were calculated. Localization precision was significantly correlated with age (*r* = +0.68) even when hearing loss was controlled (*r* = +0.46). Participants younger than 50 years of age were significantly more variable in their judgments than individuals 50 or more years old; 3.9 and 10.1°, respectively. When three levels of age (younger: 26–42; middle: 45–65; and older: 74–81) were compared, age was again found to be a significant variable. Perceptual variability for the younger, middle, and older individuals was found to be 3.7, 6.8, and 12.4°, respectively. Localization accuracy was also significantly correlated with age (*r* = +0.39). Regrettably, information about the accuracy level of different age groups was not provided.

Freigang et al. (2014) compared the ability of older (*M* = 68.1 yrs.) and young (*M* = 24.1 yrs.) adults to accurately point to the origin of an unseen sound. The accuracy of perceptual judgments was determined using a “torch” that emitted a light invisible to humans. The minimum audible angle was calculated. In each trial, three sounds were sequentially presented. Two of the stimuli can from the same location while the third originated from a different location. The task of the participant was to point at the perceived location of the deviant sound. The main effect of age was significant as well as the age*position (frontal vs. peripheral targets) interaction. Overall, young individuals were more accurate than older individuals. In addition, the minimum audible angle was approximately two times greater for older than younger individuals. This was true regardless of whether the stimulus (noise) was low-frequency (0.5 kHz) or high-frequency (3.0 kHz) centered. The correlation between age and minimum audible angle was between 0.2387 and 0.3160.

Rønne et al. (2016) likewise compared the minimum audible angles of young (*M* = 39) and old (*M* = 64) individuals. In a manner similar to that of Freigang et al. (2014), participants were exposed to three sounds on each trial and their task was to identify the origin of the deviant sound. The results are similar to those obtained by Freigang and colleagues. The minimum audible angle was notably smaller for young individuals (10°) than for older individuals (20–30°). It was also discovered that manipulation of pinna-related cues affected the accuracy and variability of responses made by older participants. Younger participants appeared unaffected by such manipulations.

## Insignificant Effects of Age on Auditory Spatial Perception

Based on the summary provided thus far, an increase in chronological age appears to adversely affect the ability of individuals to determine whether an unseen sound source is located to the right or left and to precisely locate a sound-producing object. However, as will shortly become evident, the findings of other studies as well as the findings of a number of the aforementioned studies suggest auditory spatial perception is unrelated to chronological age.

### Lateralization Perception

As mentioned previously, Fink et al. (2005) discovered that older participants needed a significantly greater time period between two sequentially presented in order to accurately determine whether a sound was located to the right or left. While the results suggested age was a significant factor affecting perception, the extent of the effect of age was dependent on the acoustic properties of the target. When the target was a sinusoidal tone, perceptual differences between younger and older participants were clearly evident. However, when the sound stimulus was a click (noise), the two age groups performed in a highly similar manner. Thus, the argument that age negatively affects sound lateralization should be promoted with qualification.

The previously mentioned study by Kołodziejczyk and Szelag (2008) also seems to support the argument that chronological age negatively affects auditory lateralization ability. While individuals in their 60s required a greater separation in time between the two sequentially present stimuli than did individuals in their 20 s, the threshold difference was not statistically significant. The suggestion that age negatively influences lateralization was only supported by the performance of centenarians. Without additional investigation, it remains possible the deterioration in performance by those over 100 years of age was a function of factors, such as cognitive processing and not audition.

### Localization Perception

With regard to sound localization, the findings of two of the earlier mentioned studies suggest a null effect of age. In addition to discovering significant differences between seven age groups, Abel et al. (2000) also discovered similarities (i.e., non-significant differences). Regardless of age, all participants were poor at locating a 0.5 kHz sound and all were highly accurate at locating broadband noise. The negative impact of age was primarily limited to the 4 kHz sound stimulus. The reported overall 7–23% decrease in accuracy that coincided with an increase in age was largely a reflection of the 4 kHz sound. If performance related to the 4 kHz sound is excluded, the overall mean difference in accuracy between the youngest (10–19 yrs. of age) individuals and those in their 60s was 6.5% for the 0.5 kHz sound and 4.0% for broadband noise. With regard to specific sounds, the mean difference in accuracy between the youngest and oldest (70–81 yrs. of age) individuals was 11.5% for the 0.5 kHz sound and 9.7% for broadband noise. One could argue the difference in error between young and old and between the youngest and oldest is not substantial. Moreover, while age is a linear and continuous variable, the observed decrease in sound localization accuracy did not decrease linearly or continuously with an increase in age. This observation suggests factors other than age or one or more moderating or mediating factors may have affected auditory localization judgments (e.g., Nambu et al., 2013; Trapeau and Schönwiesner, 2018; Bednar and Lalor, 2020).

The previously mentioned study by Dobreva et al. (2011) appears to provide incontrovertible support for the notion that an increase in age negatively affects the ability of individuals to localize sounds in terms of both azimuth and elevation. As mentioned previously, younger individuals were more accurate than middle-aged and elderly individuals in both dimensions. However, Dobreva and colleagues also discovered similarities in performance across age groups. All participants overestimated azimuth and underestimated elevation. In addition, all participants were less precise in their estimations of elevation than azimuth. Similar to Abel et al. (2000), the negative impact of age was stimulus dependent. Age was a significant factor for certain types of wideband noise (e.g., 10–20 kHz), but not for others (e.g., 3–10 kHz). Age was a null factor when the sound was low-frequency narrowband noise.

Addleman et al. (2019) compared younger (21–33 yrs.) and older (58–78 yrs.) individuals in terms of lateralization and localization performance. Comparisons were also made between centrally (10–30°) and peripherally (60–80°) located sound sources. Performance was evaluated in terms of precision (absolute error) and variability. In short, the two age groups did not significantly differ in any discernible manner. Both age groups were equally accurate (approximately 7° of error) and equally variable (again, approximately 7°). This was true regardless of whether the sound source was located centrally or peripherally.

Abel and Hay (1996) compared young-normal hearing (18–38 yrs.), old-normal hearing (41–58 yrs.), and old-hearing impaired (42–73 yrs.) individuals. Given the focus of the present paper is specifically with regard to the relationship between age and auditory spatial perception independent of hearing impairment, the performance of the old-hearing impaired group will not be discussed. The task of participants was to identify which one of an array of speakers was the origin of a target sound. The background was either quiet or continuously contained white noise. In brief, age was not found to be significant. The presence of background sound negatively affected the performance of both age groups. Variations in signal frequency (0.5 or 4 kHz) equally affected the performance of the two age groups. Age of participant was also found to be unrelated to either the accuracy of left-right judgments and front-back judgments. In a similar vein, both age groups made a comparable number of front-back and back-front reversals and both age groups were 20% more likely to make a back-to-front reversal than a front-to-back reversal.

While the focus of a study by Brungart et al. (2017) involved a comparison of young-normal hearing and older hearing-impaired individuals, the impact of age was treated, in certain circumstances, as a discrete independent variable. In that study, participants were instructed to point a handheld wand at the perceived location of an actual unseen sound source (experiment 1) or at a virtual sound source (experiment 2). Aside from the finding that an increase in angle of head movement resulted in a decrease in front-back confusions and azimuth error for both normal and hearing-impaired individuals, a stepwise regression revealed predicted localization perception was unaffected by participant age. This was true for both free-field and virtual sound localization conditions.

In a study by Otte et al. (2013), a laser pointer was affixed to the head of participants and participants were instructed to point to the laser dot at the sound source. The performance of three age groups was compared: children (*M* = 9.5 yrs.), young adults (*M* = 24.9 yrs.), and older adults (*M* = 68.4 yrs.). Judgments of target azimuth and elevation were evaluated independently. With regard to target azimuth, the three age groups performed in a highly similar manner. The correlation between actual and perceived azimuth was 0.94, 0.95, and 0.94 for children, young, and older adults, respectively. Mean absolute error was also highly similar among the different age groups. With regard to target elevation, judgments were less accurate and more variable for all three age groups. The results suggest that the accuracy of and variability in auditory spatial judgments are influenced more by concha height than by age. For all three age groups, concha height was positively related to perceptual accuracy and inversely related to perceptual variability.

Savel (2009) compared individuals varying in age on their ability to locate an unseen sound source. Loudspeakers were positioned in a 180° arc and concealed by a curtain. Participants viewed a computer screen that likewise contained a 180° arc and their task was to indicate on the computer screen the perceived location of the target sound. Perceptual accuracy was evaluated in three ways: root mean square error, standard deviation, and standard error. The results suggested perceptual abilities are unaffected by age. The correlation between age and each of the three dependent variables was not significant. Interestingly, correlations ranged from −0.32 to +0.37, which suggests an increase in age may result in either perceptual impairment as perceptual improvement. On an aside, the results further revealed left-right discrimination was unrelated to participant age.

Whitmer et al. (2021) likewise compared the ability of individuals of various ages to locate a sound. At the start of each trial, participants would hear a 5-s segment of speech from a talker located at 0° azimuth. Following a one-second delay, a speech segment from a different talker was played from a different location. The task of the participant was to turn their head and/or chair to directly face the new talker. Performance was evaluated in nine ways: trajectory start time, trajectory end time, trajectory duration, accuracy (absolute error), peak velocity, time of peak velocity, complexity, rate of misorientations, and rate of reversals. In brief, age was not significantly correlated with any of the dependent variables.

As mentioned earlier, Freigang et al. (2014) compared younger and older individuals in their ability to localize an unseen sound source. Both perceptual accuracy and minimum audible angle were calculated. With regard to perceptual accuracy, the results of various ANOVAs suggested the two age groups differed significantly. Interestingly, perceptual accuracy was not significantly correlated with participant age. This latter finding suggests perceptual judgments were influenced by a variable other than, but confounded with, age. With regard to the minimum audible angle, ANOVAs and correlation analysis suggested age was a significant factor. The authors concluded that age is not a relevant factor in terms of the ability to locate sound sources but is relevant in terms of the ability of individuals to discriminate between sound source locations.

Briley and Summerfield (2014) likewise determined the minimum audible angle of different age groups. Three age groups were compared: young (*M* = 22.9 yrs.), younger-old (*M* = 65.1 yrs.), and older-old (*M* = 76.8 yrs.). Similar to Freigang et al. (2014), participants were exposed to three pairs of sounds each separated by a period of silence. Two of the pairs originated from the same location and the third pair originated from a different location. The minimum audible angle was calculated using a 71% correct detection threshold. Although no inferential statistics were presented, the findings suggest young and younger-old adults have highly similar perceptions. When the target was located directly ahead (0° azimuth), mean MAA was 5.8° for the young participants and 6.1° for the younger-old participants. Interestingly, when the target was located in the periphery, young participants had *larger* minimum audible angles than younger-old participants. The younger-old group also seemed to be more homogenous than the young group for peripherally positioned targets. The impact of chronological age was largely limited to the performance of the older-old group. The older-old group had substantially larger minimum audible angle thresholds (8.3°) than either the young or the younger-old groups. Considering the older-old group had pronounced hearing loss, it is possible the poor performance displayed by the older-old group was a reflection of hearing loss and not age.

## Suggestions for Future Research Evaluating the Impact of Age on Auditory Spatial Perception

The summary of the research provided previously should make it clear that the relationship between chronological age and auditory spatial perception is ambiguous. Obviously, arguments can be made that the differences in findings reflect differences in methodology. Instead, I prefer to make the argument that a more accurate understanding of the effect of age on spatial perception requires an approach notably different from the one traditionally taken. More specifically, the argument will be made that an ecological approach to auditory spatial perception is required and will yield a more accurate understanding of how age affects auditory spatial judgments. Gaver (1993) argued that auditory perception experiments in general should focus on everyday listening (i.e., perception of events) rather than musical listening (i.e., perception of acoustic properties). A similar approach to that proposed by Gaver will be taken here.^2^ The subsequently offered approach will focus on the auditory perception of space, but it is equally applicable to other avenues of research. Recommendations for future research will be provided.

## Individual Factors

### Stationary Vs. Dynamic Point of Observation

In laboratory experiments, it is fairly common for observer motion to be constrained to some degree. Participants are typically seated and head movement is limited. To more effectively control changes in head position, researchers often employ a chin rest or bite bar. Limitations on head and body position are imposed for good reasons. During sound presentation, a change in head or body position can dramatically alter the sound entering the auditory canal that, in turn, can dramatically alter perception. The control of head and body movement provides an opportunity for researchers to determine the extent to which spatial judgments are influenced by factors, such as interaural level difference, interaural time differences, and various acoustic properties (e.g., frequency, intensity, and phase). Of the 16 studies previously reviewed, all 16 imposed limitations of the posture of the observers.

In real-world settings, organisms are active. We habitually change our body posture and often change our position within an environment. Rather than being passive recipients of stimulation, we intentionally seek out information and our motion makes information available when previously it was not. While humans are incapable of swiveling their ears as is commonly executed by a number of nonhumans (e.g., cats, dogs, and horses), the ears of humans are attached to a head which can be tilted and pivoted. The head is attached to a body that is capable of changing position in multiple ways (forward-backward, left-right, and up-down). By changing our position in space, we are able to detect information that could not have been detected otherwise. A number of studies have found that changes in head position have the potential to influence the judgments of sound source location (e.g., Ashmead et al., 1995; Perrett and Noble, 1997; Wightman and Kistler, 1999).

In real-world settings, individuals could become better aware of the position of a sound source by simply altering the head and/or body position. By altering the orientation of the head until interaural differences are eliminated, an observer is able to determine whether an unseen sound source is located to the right or left and they are aware of the object’s precise location. Changing one’s location within a setting also provides an opportunity for individuals to determine the change in distance of an unseen sound source. Movement that produces an increase in signal intensity at the point of observation suggests approach while movement that produces a decrease in intensity suggests withdrawal. If limitations placed on the natural response of observers potentially to yield faulty perceptual judgments, then it seems prudent to permit the individual to respond in a natural manner. Rather than limiting observer motion, researchers should permit participants to move in the manner they typically do in real-world settings. By doing so, the results obtained will better reflect the actual capabilities of individuals.

### Verbal Vs. Action-Based Tasks

Researchers investigating auditory spatial perception have traditionally required participants to verbally report target location. Participants convey their perception of target azimuth and elevation in degrees. While not discussed thus far, participants are often required to report the distance of a target using feet/inches or meters/centimeters. A clear advantage to doing so is that it permits researchers the opportunity to precisely determine perceptual accuracy. Using signed error, researchers are capable of determining the exact degree to which participants either underestimate or overestimate sound source position. Using absolute error, researchers are capable of determining the overall size of perceptual error.

Despite the advantage of employing the aforementioned response metrics, the simple fact is that it is unnatural. Individuals in real-world settings rarely, if ever, report an auditory target’s position in degrees, feet and inches, or meters and centimeters. Instead, observers typically respond to a sound. Individuals look in the direction of sudden, unexpected sounds. A telephone rings and the individual alters their gaze so that it is in the direction of the phone. A knock on the door frequently elicits the individual approaching and opening a door. As stated previously, individuals are active organisms. Empirically speaking, a number of studies have discovered that individuals are highly accurate at making action-based judgments using vision (e.g., Warren, 1984; Mark, 1987; Warren and Whang, 1987), haptics (e.g., Bingham et al., 1989; Malek and Wagman, 2008; Hajnal et al., 2020), and sound (e.g., Rosenblum et al., 1996; Russell and Turvey, 1999; Russell and Schneider, 2006).

In 1979, James J. Gibson coined the term affordance. Briefly, affordances refer to the possible actions that can be performed with an object and reflect the relationship between the perceiver and the object being perceived. Rather than estimating target azimuth in angles and distance using feet and inches, individuals performing an affordance task are required to report whether a particular action is or is not possible. Previous research supports the notion that individuals are highly capable of using sound to judge the affordances of objects (Rosenblum et al., 1996; Riehm et al., 2019) discovered that individuals are highly accurate at judging whether an object affords grasping. In fact, sound-based judgments were as accurate as those based on vision. Russell and Turvey (1999), Gordon and Rosenblum (2004), and Russell (2020) found that individuals are able to accurately determine whether a gap is large enough to afford passage. O’Neill and Russell (2017) determined that individuals are highly capable of judging whether the height of a surface affords stepping on or walking under.

In a similar manner, what one hears influences how one acts. Using only sound, individual is capable of determining when they need to act so that a moving target can be intercepted (Vernat and Gordon, 2010). What an individual hears influences their ability to maintain a stable posture (e.g., Soames and Raper, 1992; Stoffregen et al., 2009b, 2019) and the stability of their posture influences their ability to successfully use sound to judge whether a surface can be stepped upon or walked under O’Neill and Russell (2017). By acting, individuals are able to detect information otherwise unavailable. Speigle and Loomis (1993) and Ashmead et al. (1995) discovered that individuals are more accurate and less variable in their estimations of target distance when walking during the broadcast of the sound than if they were stationary. It is argued here that action responses are more natural (i.e., ecologically relevant) and incorporating such metrics in empirical investigations will yield a better understanding of how age impacts our perception of the world.

### Verbal Vs. Action Tasks

As stated previously and as was evident in the literature review, laboratory investigations commonly require participants to verbally estimate the position of a sound source and those verbal responses rarely reflect the types of actions performed in the real world. It is worth noting that when individuals make action-based judgments, what the individual says they can do does not always reflect what they actually do. For example, Warren and Whang (1987) required participants to verbally report whether the width of an adjustable doorway was sufficiently large to permit passage. The results revealed that participants required a larger gap when they actually walked through the gap than when they made verbal judgments from a stationary point of observation. Significant differences between perceptual judgments and actual performance have been documented in terms of whether a stair is low enough to be stepped upon (Warren, 1984), an object is close enough to be grasped (e.g., Carello et al., 1989; Wagman and Morgan, 2010), an expanse can be stepped or leaped over (Cole et al., 2013; Day et al., 2015), the slope of a surface is small enough for it to be ascended (Kinsella-Shaw et al., 1992), and wheelchair users can pass through a gap (e.g., Higuchi et al., 2004; Stoffregen et al., 2009a; Rodrigues et al., 2014).

While the literature suggests a disparity between perception and action, the manner by which researchers determine individual’s capabilities may be a poor reflection of what the individual can actually do. For example, measuring arm length may not accurately reflect whether an individual can grasp an object. Human observers can alter the maximum reach extent by bending forward or pivoting the torso at the waist. The findings of Butler et al. (2011), Zhong and Yost (2013), O’Neill and Russell (2017), and Wagman et al. (2019) suggest posture and postural stability affect the ability of individuals to judge accurately whether an object can be grasped. In a similar fashion, Konczak et al. (1992) determined that the maximum surface height an individual can step up on is a function not only of the individual’s leg length, but also that individual’s hip flexibility and ability to generate a sufficient amount of torque to lift the body. It should also be noted that a number of studies have discovered insignificant differences between perceptual judgments and actual performance (e.g., Mark et al., 1990; Rosenblum et al., 1996; Franchak et al., 2010).

Irrespective of whether differences exist between perception and action, and regardless of whether differences exist between an individual’s actual action capabilities and how researchers ascertain those capabilities, it would seem prudent to evaluate how individuals actually behave in real-world settings. Requiring individuals to perform an action, rather than make a verbal response, can be expected to provide greater insight into exactly how they actually perceive sound source position. For example, participants could be asked to look in the direction of a sound source. In real-world settings, individuals often direct their gaze at sound-producing objects (e.g., cell phone, orator, and animal). Likewise, participants could be asked to intercept a moving auditory target as was required by Vernat and Gordon (2010). Doing so would provide insight into the ability of individuals of various ages to accurately determine the movement of sound-producing objects. Knowing when a sound source will arrive at a particular location is useful to pedestrians attempting to cross a street when automobiles are moving in both directions. In such instances, audition, not vision, would be more helpful. Requiring participants to perform an action-based task can notably enhance the ecological validity of a study. More importantly, the use of action-based tasks provides researchers with the opportunity to more precisely evaluate the ability of individuals to be aware of the world and how age impacts that ability.

### Unidimensional Vs. Multidimensional Perception

With rare exception (e.g., Makous and Middlebrooks, 1990), laboratory investigations into auditory spatial perception involve the manipulation of sound source position along a single dimension. With respect to the perception of azimuth, targets are positioned horizontally in an arc (thereby maintaining a constant observer-source distance) and at a fixed elevation (typically ear height). With respect to the perception of elevation, targets are positioned vertically in an arc (again maintaining a constant observer-source distance) and at a fixed azimuth (the center of the arc is aligned with the observer’s midline). With respect to the perception of distance, the span between observer and target is varied, but all locations have identical elevations and azimuth is typically 0° (i.e., extending outward from with the observer’s midline). Given that researchers are often interested in the accuracy with which individuals can locate a target within a particular dimension or researchers seek to identify the information supporting perception within a particular dimension, it is advantageous to vary one dimension while other dimensions are invariant.

In real-world settings, sound sources commonly exist at different azimuths, elevations, and distance. An auditory target may be near or far, in front or behind, above or below, and at varying positions to the left or right. Rarely do targets vary along a single dimension. A change in the position of a sound source or the point of observation commonly involves sequential or simultaneous changes across multiple dimensions. Imagine the case of a stationary pedestrian whose midline is perpendicular to a road. Coincident with the approach (withdrawal) of an automobile is a decrease (increase) in distance and azimuth. An individual standing at the bottom of a flight of stairs listening to the footsteps of a pedestrian hears an object changing in distance and elevation. Rather than require individuals to judge the position of a sound source in only one dimension, future investigations in auditory spatial perception and those examining the impact of age on spatial perception may wish to explore the ability of individuals to judge the location of a sound-producing object in multiple dimensions. Independent examination of perception along different dimensions may not reflect the exact nature of how sound source position is perceived in real-world settings. Russell and Schneider (2006), for example, exposed participants to a sound source that varied in distance and azimuth and discovered that judgments were highly accurate when participants walked to the perceived location of the unseen sound and judgments were notably less accurate when participants independently reported target distance and azimuth. It is possible that, in a Gestaltian sense, the whole is different from the sum of its parts.

## Environmental Factors

### Anechoic Vs. Echoic Settings

Laboratory studies are regularly conducted in anechoic or sound-altered settings. Sound absorbing panels cover most if not all of the interior of the experimental room (floor, ceiling, and walls) or sound absorbing material is contained within the room walls. Of the 16 studies reported previously in the literature review portion of the present paper, 14 used a setting that altered sound transmission in some manner. Of the two remaining studies, one was conducted in the home of older participants due to mobility issues and one study did not provide information about the setting possibly because stimuli were presented through headphones. Auditory researchers often minimize or eliminate reverberant sound since reverberations have the potential to influence auditory spatial judgments (e.g., Mershon and King, 1975; Mershon et al., 1989; Bronkhorst and Houtgast, 1999; Zahorik et al., 2005). In order for researchers to maintain control over the acoustic information participants are exposed to, it is beneficial to minimize or eliminate reverberant sound.

Though open or free-field environments exist in natural settings, they should be considered a limiting case (i.e., abnormal). Spaces that produce little or no reverberant sound are highly uncommon in the real world. Instead, individuals commonly inhabit locations whose surfaces (floor, ceiling, and walls) reflect sound. Moreover, natural settings often contain surfaces composed of different materials (e.g., concrete, tile, and carpet) each of which can uniquely alter the sound reaching the point of observation. Therefore, it is possible the sound stimulating the ear is notably more complex and different from the sound at the origin. Regardless, it is common for perception and action to occur within settings that contain both direct and reverberant sound and previous research suggests sound reverberations significantly affect auditory spatial perception. Reverberant sound has been shown to alter sound localization judgments (e.g., Hartmann, 1983; Rakerd and Hartmann, 1985; Giguère and Abel, 1993) and the ability to detect and perceive the distance of walls (e.g., Griffin, 1944; Supa et al., 1944; Worchel and Dallenbach, 1947; Kellogg, 1962; Rosenblum et al., 2000). Ashmead et al. (1998), for example, found that observers rely on reverberations in order to walk down a corridor without colliding with the walls.

Despite the difficulty of ascertaining the change in sound that results from the presence of sound reflecting surfaces, future investigations should consider the extent to which individuals can accurately detect the position of an unseen sound source in echoic settings. Knowing how individuals of different ages estimate the position of a sound-producing object in an anechoic space may provide little to no insight into how they perceive sound source position in natural settings. Conducting research in environments commonly inhabited by individuals will provide researchers with a more accurate understanding of the information observers employ when determining sound source position and the extent to which age influences spatial perception.

### Uncluttered Vs. Cluttered Setting

The creation of a setting free from reverberation requires the elimination of clutter. Here, clutter refers to objects other than the object that is the target of perception. Laboratory settings often exclude objects not directly necessary for performing an experiment. Experimental settings seemingly contain nothing more than a participant, chair, loudspeaker(s), materials to position the loudspeaker(s), and equipment related to the broadcast of sound. The extent to which previous research has utilized a clutter-free setting is often impossible to determine from descriptions provided in published papers. Aside from describing the experimental setting as anechoic, semi-reverberant, or echoic, authors rarely include information that would permit an understanding of the extent to which the experimental setting is cluttered. Often, the reader assumes clutter has been minimized or eliminated. The minimization and elimination of clutter exist for theoretical reasons. Clutter has the potential of creating reverberant sound that, as mentioned earlier, could dramatically alter the sound reaching the ear. In short, clutter has the potential to alter observer perceptions of sound source location.

Despite the advantages associated with the absence of clutter, real-world settings are rarely clutter free. Everyday settings regularly contain numerous and various objects that are unrelated to the task. The inclusion of clutter allows researchers to investigate auditory spatial perception, and its relationship to chronological age, in a situation akin to that which individuals normally find themselves. The use of cluttered settings enhances the ecological validity of studies and provides insight into the extent to which individuals accurately locate objects in everyday settings. As stated earlier, the inclusion of clutter may provide participants with information (e.g., reverberant sound) that enhances perceptual judgments.

The use of a cluttered setting also provides new avenues of research. A single piece of clutter could either obstruct or occlude a sound-producing object. A sound source is deemed obstructed if the object is small enough that sound can travel around it. If the object is large enough to fully block sound transmission, the sound source is deemed occluded. In real-world settings, individuals are commonly exposed to obstructed or occluded sound-producing objects. Despite the presence of the walls of my office, I can easily use sound to detect the existence of a pedestrian and I am able to detect the direction of their motion (approaching or receding). Based solely on what I hear, I am keenly aware of whether a colleague who is speaking to me is located in my office doorway or their adjacent office. Recently, Russell and Brown (2019), Gordon and Rosenblum (2004), and Kolarik et al. (2016) reported that individuals are highly capable at both detecting and creating occlusion. Given that clutter is common in natural settings, future investigations may wish to include occlusion and/or obstruction, for example, in their experimental designs when investigating auditory spatial perception and when determining the extent to which chronological age affects spatial judgments.

## Acoustic Factors

### Simple Vs. Complex Sounds

Laboratory experiments investigating the impact of age on judgments of sound source position typically involve simple sounds. Out of the 16 studies reviewed earlier in this paper, 15 used pure tones or noise stimuli. Only one study used a complex sound stimulus (a speech segment). Simple sounds are sounds whose acoustic properties (e.g., frequency and amplitude) remain unchanged over time. Examples include musical notes, pure tones, and noise (e.g., white noise, pink noise, and brown noise). The use of simple sounds in laboratory experiments is expected given that signal frequency affects interaural level differences and interaural temporal differences. It is well known that an increase in frequency results in greater interaural level (intensity) differences and that a decrease in frequency results in greater interaural temporal differences. Changes in signal frequency result in changes in interaural differences that, in turn, result in changes in azimuth perception. With respect to auditory distance perception, while signal intensity is clearly a relevant factor, so is signal frequency. Changes in signal frequency have been shown to influence distance judgments (e.g., Blauert, 1997; Brungart, 1999; Kopčo and Shinn-Cunningham, 2011) and the ability of individuals to detect changes in sound level (e.g., Riesz, 1933; Jesteadt et al., 1977), which occurs with a change in actual distance. More specifically, the loss of higher frequency components yields the perception of a more distant sound source. Knowing that signal frequency affects distance perception, it should come as no surprise that the alteration of signal frequency affects the perception of approach or withdrawal. Gordon et al. (2013), for example, determined that a decrease in the center frequency of noise bands resulted in an increase in the accuracy of estimations of sound source arrival. In sum, the use of simple sounds provides researchers with an opportunity to examine the extent to which particular acoustic properties affect spatial perception.

In the real world, simple sounds are rare and complex sounds are highly common. Complex sounds are sounds whose acoustic properties (e.g., frequency, amplitude, amplitude envelopes, and onset and/or offset) vary over time. The vast majority of everyday events (e.g., a door closing, footsteps, and rain hitting the ground) creates complex auditory events. While advantages exist for using simple sounds, the lack of naturally occurring changes in acoustic structure may deprive participants of information necessary to accurately judge sound source location. Schutz and Gillard (2020) reviewed 443 articles and discovered that only 11% of 1,017 of the reported experiments used a dynamically varying sound. While flat tones are more common in laboratory settings, percussive tones are more representative of the sounds individuals normally encounter in real-world settings and, thus, are more often the sounds individuals judge and respond to. The inclusion of a sound that changes in structure has the potential to affect one’s perception of the world. Individuals who watched two disks moving toward one another were vastly more likely to report the disks bouncing off one another if a click sound occurred at or near time of collision (Sekuler et al., 1997). More relevant to our discussion of complex sounds, Grassi and Casco (2009) discovered that the disks were perceived as bouncing off one another if a ramped sound (increasing intensity) occurred at the moment of contact. Damped sounds (those decreasing in intensity) created the impression the disks passed through one another. With regard to auditory spatial perception, Rakerd and Hartmann (1986) and Hartmann and Rakerd (1989b) found that the extent of signal onset and offset notably affected the ability of individuals to accurately determine the origin of a sound. These studies and others suggest that future investigations into the influence of age on perception of sound source location should determine the extent to which variations in dynamic sounds affect perceptual reports. The use of dynamic stimuli will serve to enhance our understanding of how it is that we perceived the world.

### Brief Vs. Extended Sounds

It is incredibly common for researchers to utilize brief sounds when determining the extent to which chronological age impacts auditory spatial judgments. Of the 16 studies presented earlier, mean stimulus duration was 630.8 ms and only two studies used a stimulus that lasted more than 1 s. If the two exceptional studies are excluded from consideration, mean stimulus duration was a very brief 227.1 ms. The use of brief stimuli can be expected to reduce participation time and therefore minimize or prevent participant fatigue. However, I am unaware of any published papers that advocate the advantage of using brief sounds.

Though researchers commonly use brief stimuli, auditory events in real-world settings commonly last for extended periods of time. The sound of a pedestrian, an automobile, and a conversation, for example, persist for several seconds. Akin to the notion that a Fourier analysis transforms a complex sound into a conglomeration of simple sounds, the argument can be made that auditory events are nothing more than a composite of brief and sometimes repetitive sounds. The sound of a pedestrian, for example, can be considered as nothing more than a collection of a number of discrete and individually identifiable footsteps. Nonetheless, the counterargument can be made that the information serving as the foundation of auditory spatial judgments is the entire event. It is possible even slight differences between repetitive sounds and/or repeated exposure to the same sound may affect spatial perception. As discussed by Gaver (1993), the real world is composed of auditory events that exist over time and it is the entirely of the event that individuals perceive. Empirically speaking, stimulus duration affects auditory spatial judgments. More specifically, a decrease in stimulus duration has been shown to result in a decrease in perceptual accuracy (e.g., Macpherson and Middlebrooks, 2000; Vliegen and Van Opstal, 2004; Yost and Pastore, 2019). For both theoretical and empirical reasons, it is suggested that future investigations into the influence of age on auditory spatial perception should involve prolonged sounds and, if applicable, complete auditory events. By taking such an approach, it is expected our findings will more accurately reflect individual perception as it occurs in real-world settings, have enhanced ecologically validity, and yield a better understanding of how we perceive the world and how age influences those perceptions.

### Stationary Vs. Dynamic Sound-Producing Objects

In addition to brief stimuli, investigations into the relationship between age and spatial perception typically involve auditory targets that are stationary during sound presentation. Of the 16 studies presented earlier, all 16 involved stationary sound sources. The use of stationary sources is advantageous since it provides an opportunity for researchers to determine the accuracy with which individuals can determine the location of a target. For example, Mills (1958) and Voss et al. (2004) determined that, depending on the frequency of the stimulus, the minimum audible angle was as low as 1–3°. Oldfield and Parker (1984) determined that the mean absolute error in terms of azimuth was 9° while mean absolute error in elevation was 12°. Similarly, Makous and Middlebrooks (1990) found mean error to be 2° for azimuth and 3.5° for elevation. Use of stationary sound sources also provides researchers with the opportunity to determine the extent to which various acoustic features (e.g., frequency, onset/offset, and intensity) influence spatial judgments. Since a change in observer-source position could dramatically alter the sound contacting the ear, it is imperative to hold source position constant.

As in laboratory settings, real-world settings often contain stationary sound-producing objects. The distance, azimuth, and elevation of a ringing telephone are constant unless acted upon by the individual. The same can be said for a wide variety of everyday objects (e.g., television, computer, and dishwasher). Nonetheless, real-world settings also contain sound sources that are in motion (e.g., motor vehicles, pedestrians, and birds). Use of dynamic sound sources (i.e., sound sources whose position changes over time) allows researchers to investigate the types of spatial judgments individuals frequently make in everyday settings; the kind of judgments not possible with a stationary source. Rosenblum et al. (1987, 1993), Schiff and Oldak (1990), and Silva et al. (2017) found individuals often and somewhat poorly estimate when a sound-producing object will arrive at their location. Neuhoff (1998, 2001) discovered individuals are apparently more sensitive to approaching than receding auditory objects. Neuhoff (2016) further discovered approaching sounds are perceived as moving faster than receding sounds. Recently, Russell and Herl (2021) discovered individuals are capable of distinguishing between auditory events that involved hard (forceful) and soft (gentle) contact. In addition to being ecologically valid, the use of dynamic sound sources provides an opportunity to explore new avenues of research that, in turn, will expand our understanding of how chronological age affects auditory spatial judgments.

## Summary

Two purposes existed with respect to the present paper. First, the author wished to review the research investigating the relationship between chronological age and auditory spatial perception. What follows is a brief summary of the findings:

- When examined independently of hearing impairment, only a relatively small number of studies have investigated the degree to which age influences auditory spatial perception.
- Roughly, an equal number of findings suggest an increase in age either adversely affects spatial perception or has little or no influence.
- The adverse effect of age on the perceived sound source location appears to be dependent on a variety of factors (e.g., signal frequency, presence of background noise, and centrally or peripherally located sources).
- Notable differences exist between studies in terms of experimental design (e.g., stimuli, task, and definitions of “older”) which may or may not account for differences in findings.
- While the relationships between age and azimuth perception and age and elevation perception have been examined, it appears no known study has examined the impact of observer age on auditory distance perception.

Based on the literature, it is not yet possible to render a firm conclusion about the relationship between age and spatial perception. Additional research is clearly needed.

A second purpose of the present paper was to compare how auditory spatial investigations, including but not limited to those examining the impact of age, are often conducted in laboratory settings and the manner with which individuals perceive sound source position in real-world settings. It is hoped the comparison between laboratory and everyday settings is not interpreted as a criticism of previous research. When possible, the author has presented a rationale as to why experimenters have utilized particular settings, sounds, and methods. As stated throughout the paper, solid theoretical reasons exist for why research has been conducted in the custom it has been. The sole intention of the author is to simply provide alternatives to the traditional approach; alternatives that are based on the environments commonly inhabited by observers, the sounds they are frequently exposed to, and the manner with which they normally respond. What follows is a summary of the notable differences that exist between the laboratory environment and everyday settings:

- Laboratory research commonly involves stationary individuals making verbal judgments of a stationary sound source. Judgments are made in an anechoic, uncluttered setting. With rare exception, verbal judgments reflect one dimension. Sound stimuli are typically brief and simple in nature.
- In everyday settings, individuals are commonly exposed to auditory events that exist for extended periods of time and are complex in acoustic structure. Those sounds occur in echoic, cluttered spaces and can originate at any point across multiple dimensions. In response to a sound, individuals often alter the position of the head and/or body and either make an action-based judgment or behaviorally react to a sound-producing object. The sound-producing object can be stationary or dynamic.

In brief, numerous and vast differences exist between laboratory and real-world settings.

It is the belief of the author that, despite solid theoretical reasons, laboratory research is largely lacking in ecological validity. Research conducted in an ecologically valid manner permits researchers to investigate auditory spatial perception as individuals do in everyday settings. It is believed that an increase in ecological validity will yield results that more accurately reflect the abilities of individuals to judge the position of a sound-producing object. Using a more natural design also provides researchers with an opportunity to better determine the extent to which chronological age influences spatial judgments. Furthermore, research conducted in an ecologically relevant manner provides new avenues for research (e.g., perception of occluded sound sources). Those avenues have direct bearing on what individuals actually do in everyday settings. Finally, it is likely that ecologically based research will have greater external validity and greater construct validity in comparison with research conducted in the traditional manner. Clearly, additional research is needed to confirm or discredit the arguments presented here.

## Author Contributions

The author confirms being the sole contributor of this work and has approved it for publication.

## Conflict of Interest

The author declares that the research was conducted in the absence of any commercial or financial relationships that could be construed as a potential conflict of interest.

## Publisher’s Note

All claims expressed in this article are solely those of the authors and do not necessarily represent those of their affiliated organizations, or those of the publisher, the editors and the reviewers. Any product that may be evaluated in this article, or claim that may be made by its manufacturer, is not guaranteed or endorsed by the publisher.

## Footnotes

^1^Humans and most nonhumans are capable of localizing unseen sound sources despite substantial differences between species. With regard to humans, the size and structure of the head are such that sound either travels around it or is blocked by it (thus, creating what is referred to as acoustic shadow). In several species (e.g., fish, amphibians, and reptiles) where tissue density is substantially lower, sound travels through the body, head, or mouth. In humans and other species, the ears are physically separated in space, which provides an opportunity to determine sound source position using interaural differences. In other species (e.g., birds), the ears are physically and internally coupled by an interaural canal thereby making interaural differences moot. Additional physical differences between species (e.g., form of the tympanic membrane, presence of the three bones of the middle ear, and presence and shape of the pinna) as well as significant differences in experimental methodology make comparisons between species difficult. For these reasons, the present paper will focus on the abilities of humans of various ages to determine the position of a sound-producing object. However, interspecies comparisons suggest the existence of lawful relationships. Extent of high-frequency hearing appears to be related to the distance between the ears. Sound localization acuity is inversely related to the breadth of an animal’s field of best vision. A number of publications discuss interspecies and intraspecies differences and similarities with regard to auditory spatial acuity (e.g., Heffner and Heffner, 1998, 2014, 2016, 2018).

^2^The reader may note that the ecological approach discussed in the present paper resembles the ecological approach proposed by Gibson (1979) and wonders why the Gibsonian approach was not discussed in more depth. First, I intend to subsequently submit a paper that directly contrasts¸ with regard to auditory spatial perception, the traditional and Gibsonian, ecological approaches. That paper will delve more deeply into the Gibsonian approach and include discussion of concepts, such as affordances, effectivities, mutuality, and prospective control. Second, while the Gibsonian, ecological approach could have been included in the present paper, I do not believe the Gibsonian, ecological approach can be completely and accurately presented in a brief manner, as would have been required here. I believe the contrast between the traditional and Gibsonian approaches should be the sole focus of a separate paper and considered independently of the relationship between chronological age and auditory spatial perception.
